# Sex-Specific Survival, Growth, Immunity and Organ Development in Preterm Pigs as Models for Immature Newborns

**DOI:** 10.3389/fped.2021.626101

**Published:** 2021-02-11

**Authors:** Ole Bæk, Malene Skovsted Cilieborg, Duc Ninh Nguyen, Stine Brandt Bering, Thomas Thymann, Per Torp Sangild

**Affiliations:** ^1^Comparative Pediatrics and Nutrition, Faculty of Health and Medical Sciences, University of Copenhagen, Frederiksberg, Denmark; ^2^Department of Neonatology, Rigshospitalet, Copenhagen, Denmark; ^3^Department of Pediatrics, Odense University Hospital, Odense, Denmark

**Keywords:** sex, gender, preterm, immune, animal model, cohort

## Abstract

**Background:** After very preterm birth, male infants show higher mortality than females, with higher incidence of lung immaturity, neurological deficits, infections, and growth failure. In modern pig production, piglets dying in the perinatal period (up to 20%) often show signs of immature organs, but sex-specific effects are not clear. Using preterm pigs as model for immature infants and piglets, we hypothesized that neonatal survival and initial growth and immune development depend on sex.

**Methods:** Using data from a series of previous intervention trials with similar delivery and rearing procedures, we established three cohorts of preterm pigs (90% gestation), reared for 5, 9, or 19 days before sample collection (total *n* = 1,938 piglets from 109 litters). Partly overlapping endpoints among experiments allowed for multiple comparisons between males and females for data on mortality, body and organ growth, gut, immunity, and brain function.

**Results:** Within the first 2 days, males showed higher mortality than females (18 vs. 8%, *P* < 0.001), but less severe immune response to gram-positive infection. No effect of sex was observed for thermoregulation or plasma cortisol. Later, infection resistance did not differ between sexes, but growth rate was reduced for body (up to −40%) and kidneys (−6%) in males, with higher leucocyte counts (+15%) and lower CD4 T cell fraction (−5%) on day 9 and lower monocyte counts (−18%, day 19, all *P* < 0.05). Gut structure, function and necrotizing enterocolitis (NEC) incidence were similar between groups, but intestinal weight (−3%) and brush-border enzyme activities were reduced at day 5 (lactase, DPP IV, −8%) in males. Remaining values for blood biochemistry, hematology, bone density, regional brain weights, and visual memory (tested in a T maze) were similar.

**Conclusion:** Following preterm birth, male pigs show higher mortality and slower growth than females, despite limited differences in organ growth, gut, immune, and brain functions. Neonatal intensive care procedures may be particularly important for compromised newborns of the male sex. Preterm pigs can serve as good models to study the interactions of sex- and maturation-specific survival and physiological adaptation in mammals.

## Introduction

Across the lifespan, overall morbidity and mortality is higher in males than in females, caused by a multifaceted interaction among biological factors, societal conditions, and environmental determinants (1). The physiological mechanisms of the interacting sex- and age-related morbidities remain unknown and studies indicate that the male deficits are greatest at short gestational age at birth (2). Males are overrepresented among preterm infants and show an increased post-natal mortality compared with their female counterparts (3). Male sex is more prevalent with decreasing gestational age at birth, suggesting that male sex itself may be a risk factor for preterm birth (4). After preterm birth, deficient respiratory and immune functions may increase mortality (4, 5), potentially linked to later reduced body growth (6, 7) and more frequent neurological sequelae (4). While such complications may be specific for preterm birth, indications of increased mortality of term male infants, across the entire lifespan, imply that differences in survival capacity may exist between sexes, even after term birth (1, 8).

It is unknown if neonatal survival and adaptation is sex-specific across mammalian species. For pigs, perinatal mortality is high (15–25%) in both modern and traditional farming systems (9–15). A higher male piglet mortality is reported for outdoor, extensive systems with moderate litter size (16, 17) and impaired locomotion, hypothermia and lacking energy and passive immunity via sow's colostrum have been suggested as causative factors for increased male mortality (16). Intensive breeding programs for prolonged lean tissue growth (physiological immaturity) and large litter size in high-intensity facilities may increase the number of weak piglets (18–20). Considering the above knowledge of sex-specific effects for preterm infants, this may increase sex-specific neonatal morbidities for modern piglets. Pig growth rates and litter size are top in the world in Denmark (mean 18, range 15–30 piglets/sow) and perinatal mortality remains high, despite genetic selection for survival (9, 21). Previous studies in preterm pigs suggest that the combination of immaturity and growth restriction at birth negatively affects systemic and gut immunity (22–25).

Among the immune-related morbidities in preterm infants, necrotizing enterocolitis (NEC), a serious gut inflammatory disorder (26), does not appear to differ between males and females, although few studies report higher NEC incidence in males (27). Conversely, sex-specific differences in systemic immunity complications are reported, and full-term male infants show weaker innate and adaptive immunity, reduced vaccine response and poorer pathogen clearance (8, 28). These results indicate fundamental sex-specific differences in systemic immune functions, possibly driven by sex hormones, because differences accelerated after puberty in the above studies. Other hormones, such as glucocorticoids, critical for neonatal maturation and survival across many species, could also play a role for sex-specific survival after term birth (29, 30). Less is known for immature infants, but because sex-specific differences may manifest themselves already *in utero* (31), it is plausible that immaturity at birth pre-dispose to sex-specific effects on morbidity and mortality. Further, maternal inflammation and infection are known to affect infant immunity both in the neonatal period and beyond (32, 33). Male term and preterm infants also have higher risk of positive blood cultures and sepsis, indicating a higher post-natal sensitivity to infection (4, 34), and cord blood from male infants show greater pro-inflammatory response to lipopolysaccharide (LPS) (35). However, in other studies cord blood mononuclear cells from term and preterm infants did not show sex-specific differences in response to Toll-like receptor agonists (36). While these data confirm sex-specific responses and morbidities in some, but not all, human studies, they do not provide insight into mechanisms and whether sex effects exist across mammals with/without preterm birth. Observational studies in preterm infants provide limited insight into organ-specific mechanisms and results are often confounded by variable fetal conditions, gestational ages and post-natal treatments.

During 20 years, we have conducted numerous experiments with preterm pigs as models for preterm infants and immune-compromised newborn production pigs, using similar procedures for delivery (elective cesarean section at 90% gestation) and neonatal care (e.g., incubator rearing with supplemental oxygen, heating, and parenteral/enteral nutrition) (37, 38). This animal model has been used to assess effects of immaturity itself (reduced gestational age at birth) (39–45) and dietary, microbial and pharmacological interventions on nutritional (46), gastrointestinal (47, 48), immune (49, 50), and neurological endpoints (51–54). Across these separate experiments, no consistent sex-specific effects were reported. Larger cohorts of preterm pigs, across variable clinical complications and interventions, may be required to demonstrate sex-specific effects. We hypothesized that cohorts of preterm pigs, like preterm infants, show increased mortality of male offspring, potentially related to sex-specific development of organ growth and gut, immune and brain functions. This knowledge may help to define the need for sex-specific care procedures in pig production (e.g., intensive care procedures, artificial rearing, cross-fostering) as well as in human neonatatology.

## Methods

### Animals and Their Treatment

All animal experiments were conducted under a license from the Danish National Committee on Animal Experimentation (2014-15-0201-00418). We compiled a database from previous experiments performed with preterm pigs 2009-2020, all using the same delivery procedures and rearing facilities (37). For all measured outcomes in total 1,938 preterm pigs from 109 litters across 37 experiments (Table 1), we collected sex-specific results when the same outcome parameter was measured across several experiments (aiming to have *n* > 100 for each sex). Across the different experiments, preterm pigs were reared for 4–5, 8–9, or 19 days and the three cohort groups were denoted 5, 9, and 19 day cohorts. An overview of the number of pigs, litters and experiments with specific interventions related to diet (e.g., feeding of formula, porcine, bovine or human milk, or colostrum), microbes (e.g., administration of pre-, pro- and antibiotics) or drugs (e.g., cortisol, IGF-1) is shown in Supplementary Table 1. While some biological endpoints were shared among experiments, other parameters were assessed only in some experiments, resulting in different n numbers for different parameters for each cohort. For immune endpoints, we first assessed the sex-specific responses to systemic infection for subgroups of pigs younger than 5 days, not included in the 5, 9, or 19 day cohorts (described in detail later).

**Table 1:** Overview of cohorts of preterm pigs.

**Duration (days)**	**Preterm pigs total *n***	**Litters *n***	**Separate studies *n***	**Females *n***	**Males *N***
5	1,398	79	27	698	700
9	319	18	5	163	156
19	221	12	5	104	117

**Figure 1:**
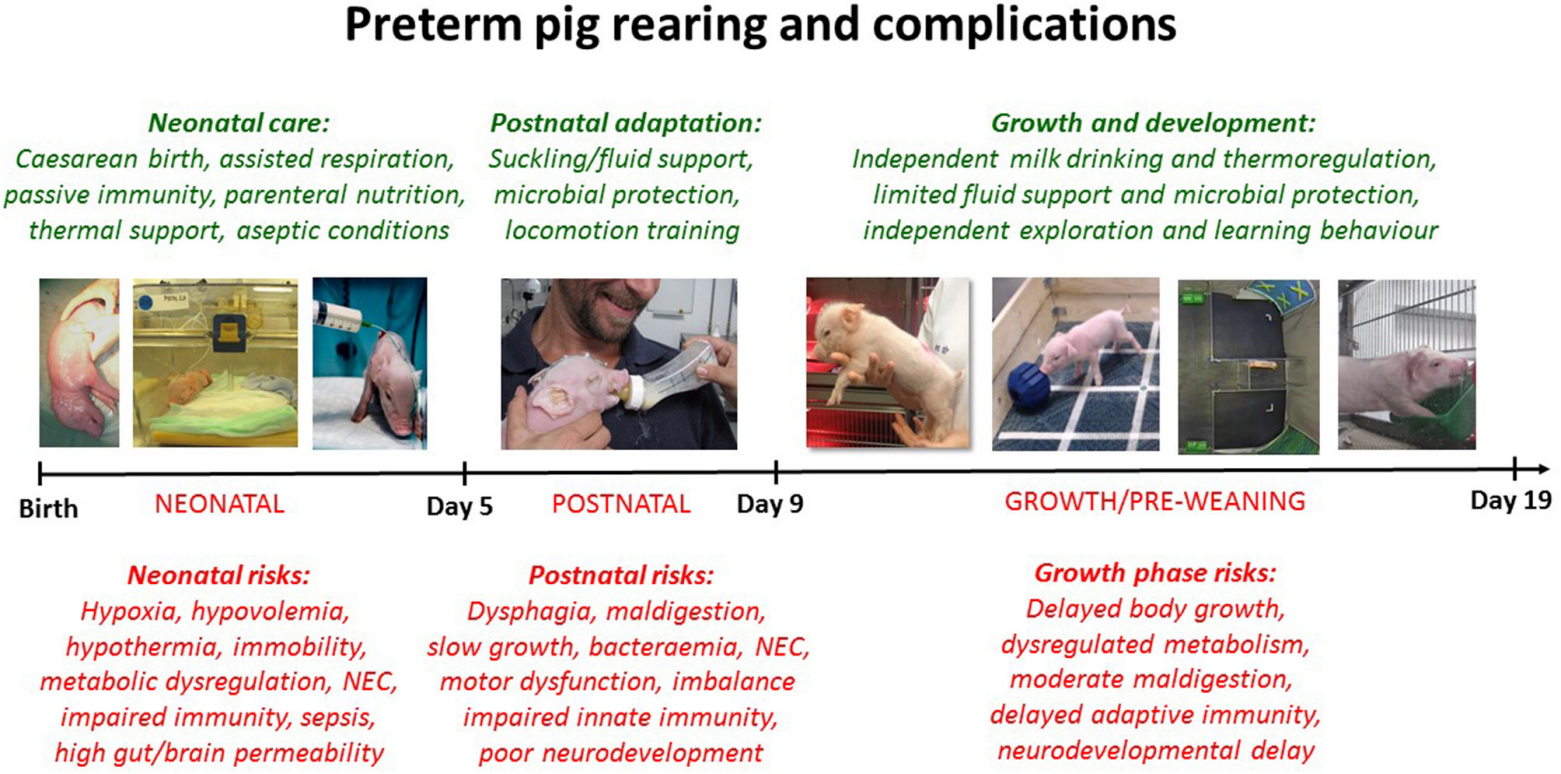
Illustration of clinical care procedures (green text) and possible morbidities (red text) for cesarean-delivered 90% gestation preterm pigs, reared as models for preterm infants. Preterm pigs show clinical and physiological characteristics reflecting very preterm infants (<32 weeks gestation) but comparisons to infants are both age- and organ-specific (37). Based on the reports of gender-specific morbidities in preterm infants, we investigate if sex-specific effects are present in preterm pigs at different stages after preterm birth.

All pigs (Duroc × Yorkshire × Danish Landrace) were delivered by elective cesarean section at 105–106 days gestation (term = 117 ± 1 days), and while anesthetized, piglets were fitted with oro-gastric feeding tubes and umbilical arterial lines. The pigs were reared in incubators with supplemental oxygen for the first 12 h (1–2 L/min), extra heating to prevent hypothermia and standardized parenteral and enteral feeding, as described previously (37). In the day 5 cohort, pigs had their rectal temperature taken at 2 h intervals for the first 12 h of life (data available for *n* = 719, 49% male). Furthermore, all pigs were infused with maternal plasma (16–20 mL/kg) within the first 24 h to ensure a standardized level of systemic passive immunity to support immunological protection (e.g., maternal IgG), independent of their sow's colostrum, thus excluding by this artificial rearing system any variability induced by differential piglet-sow interactions. Figure 1 presents an overview of rearing conditions and possible clinical complications in 90% gestation preterm pigs when reared for 5, 9, or 19 days.

Several different enteral diets and interventions were used in the studies (see Supplementary Table 1) while the same formulation of parenteral nutrition (modified composition of Kabiven, Fresenius Kabi, Sweden) was used across experiments (41, 48, 51, 55). Across all experiments, pigs were randomly allocated to treatment groups stratified by sex and birth weight, thereby ensuring an even sex distribution in each intervention. During the studies, pigs were euthanized ahead of time if serious complications developed, defined as humane endpoints in accordance with criteria and the license from the Danish National Committee on Animal Experimentation. As described previously (37), the majority of mortalities for preterm pigs reared under such conditions occur within the first 48 h of life. Therefore, only pigs dying within this early neonatal period were included into mortality data for the present study. Pigs dying from iatrogenic causes (e.g., catheter-related complications with blood loss) were excluded from the analyses. At the end of the pre-defined study periods, all pigs were sacrificed by intracardial injection of phenobarbital after which organ weights were recorded and tissues sampled according to the different study protocols.

### Neonatal Mortality and Blood Immunity, Hematology and Biochemistry

Across all experiments, we identified the litters where mortality within the first 48 h was accurately reported and noted as spontaneous death or euthanasia. Respiratory distress was commonly observed for such preterm pigs, but a detailed clinical and post-mortem organ investigation of the piglets was not performed. Hematology parameters were evaluated at birth (from the cord, during cesarean delivery), day 9 and day 19 (Advia 2120 Hematology System, Siemens Healthcare Diagnostics, Tarrytown, NY, USA) and plasma biochemistry was recorded at day 19 (Advia 1800 Chemistry System, Siemens, Erlangen, Germany).

Before analyzing organ data for the 5, 9, and 19 day cohorts, we explored the sex-specific differences in neonatal immune response by re-examining data from two previously conducted experiments modeling neonatal sepsis in infants (23, 56). These animals were not included among the 5, 9, or 19 day cohorts because the study length was shorter than 5 days. In short, preterm pigs were infused with live *Staphylococcus epidermidis* (1 × 10^8^-5 × 10^9^ CFU/kg body weight) bacteria through the umbilical caterer, either few hours after birth (*n* = 38, 53% male) or after 48 h (*n* = 39, 56% male), without prior provision of maternal plasma. The animals were followed for 24–48 h and hematological and arterial blood gas parameters evaluated. Animals inoculated with bacteria at birth were kept exclusively on parenteral nutrition whereas those inoculated after 48 h were supplemented with enteral milk diets. A detailed description of the experimental setup and bacterial inoculation procedure is available (23).

In the 9 day cohort, and in a subgroup of the 5 day cohort (*n* = 75, 40% male), spontaneous bacterial infection of the bone marrow was determined. After euthanasia, the femur head was dissected in a sterile manner and a sample of bone marrow collected. This sample was homogenized, serially diluted, plated out on agar and cultured for 24 h. Afterwards, bacterial density was calculated as culture-forming units (CFUs) per milliliter of bone marrow homogenate.

In 9 day pigs, and in a subgroup of day 19 pigs (*n* = 148, 51% male), flow cytometry (FACS) was used to determine T cell subsets, as described elsewhere (24). Using fluorescent-labeled antibodies against CD3, CD4, CD8, and FOXP3, the fraction of T cells, CD4+ T cells, CD8+ T cells and regulatory T cells (CD3+CD4+FOXP3+) were established. For the same pigs, the FACS equipment was used to determine neutrophil phagocytic function (57). In short, whole blood samples were incubated with fluorescent-labeled *Escherichia coli* (pH rhodo, Thermofischer, USA) and the phagocytic rate was defined as the fraction of neutrophils with internalized bacteria and the phagocytic capacity as the median fluorescent intensity of those neutrophils.

In the 9 day cohort, leucocyte gene expression at birth was evaluated using cord blood. Using primers against a panel of immune related genes, the relative expression of genes in whole blood, before and after stimulation with LPS, was calculated. Gene expression levels were presented as fold change, relative to a housekeeping gene. The same analysis was repeated at day 9 for a subgroup of animals (*n* = 38, 53% male). A full description of the genes investigated and methodologies are published elsewhere (22). Cortisol levels in plasma were measured by enzyme linked immune assay (R&D systems, USA) in cord blood (*n* = 112, 49% male) and at euthanasia in the day 5 (*n* = 164, 44% male) and day 19 cohorts (*n* = 60, 53% male).

### Growth, Organ Weights and Gut Endpoints

Body weights at birth and euthanasia were used to calculate growth rate as relative daily weight gains across the study period (g/kg/day). At euthanasia, all major internal organs were evaluated and weight relative to body weight recorded. In a subgroup of the 19 day cohort (*n* = 86, 53% male) a full body dual-energy X-ray absorptiometry (DEXA, Lunar Prodigy scanner, GE Healthcare, Little Chalfont, UK) was performed at euthanasia to determine body composition, as described previously (41).

Sensitivity to NEC in preterm pigs is highest during the first 1–2 weeks after birth (58), and for the 5 and 9 day cohorts, the stomach, small intestine, and colon was visually inspected post-mortem for signs of inflammation and occurrence of NEC lesions, according to the same validated scoring system, with score 1–2 representing healthy tissue, 3–4 some evidence of NEC lesions, and 5–6 reflecting severe lesions (59). Brush border enzyme activities, including sucrase, maltase, lactase, aminopeptidase N (ApN), aminopeptidase A (ApA), and dipeptidyl peptidase IV (DPPIV), were evaluated across the small intestine for the 5 and 19-day cohorts (48). Using formalin-fixed small intestinal tissue, villus height and crypt depths were determined (48).

### Neurodevelopment and Behavior

Brain weights were recorded and specifically for the 19 day cohort, the brains were further dissected to assess the relative size of each brain region [as percentage of the whole brain (60)]. Furthermore, in the 19 day studies, we performed a T maze-test, as a measure of spatial memory (Figure 1), explained in detail elsewhere (51, 52, 54). Briefly, from 10 days of age preterm pigs were placed in a T-shaped maze with a milk reward in one arm. Using visual clues on the walls, pigs could learn to find the reward following their daily tests in the maze. The number of days until pigs chose the right path in at least 80% of trials, was considered as the time taken to learn the task. The pigs were also subject to an open field test investigating explorative free movement behavior [Figure 1, (54)].

### Statistical Analyses

All statistical analyses were performed using Stata 14.2 (StataCorp, Texas, USA). Categorical data was compared by Fischer's exact-test and an unadjusted odds ratio, with corresponding confidence interval (CI) calculated. Continuous data were compared using a linear mixed effect model with litter and diet type as fixed factors. Variables that could not conform to normal distribution were logarithmically transformed. If normal distribution could not be obtained, data was compared by Kruskal Wallis'-test. Data collected at several time points were mostly from independent samples and therefore compared separately for each time point. When analyzing immune related endpoints any animals treated with an antibiotic intervention were censored from analysis. Results were presented as means with corresponding standard error of the mean (SE).

## Results

### Neonatal Mortality and Blood Immunity, Hematology and Biochemistry

Across the 109 litters of pigs, there were a mean of 17.8 live-born piglets per sow (Table 1). Mortality within the first 48 h was recorded for 833 live-born preterm pigs (48% male) from 18 experiments and 44 of these litters. From these litters, 107 (13%) died within 48 h of birth. The mortality was higher in male than female piglets (18 vs. 8%, *P* < 0.001). The unadjusted odds ratio for male piglets dying within 48 h compared to female was 2.4 (CI: 1.6–3.8, *P* < 0.001). Rectal temperatures decreased in the first 3–4 h after birth, despite being reared in incubators (to 34–35°C), but recovered thereafter to normal temperatures, as shown previously for preterm vs. term pigs (61). The temperature curve during the first 24 h after birth did not differ between male and female pigs (data not shown).

Hematological parameters at birth did not differ between female and male preterm pigs. By day 9, male piglets had higher total leucocyte counts (Table 2, *P* < 0.05) with a tendency to lower hemoglobin and haematocrit values (Table 2, both *P* = 0.09). At day 19, the male piglets showed lower hemoglobin concentration and haematocrit values (Table 2, both *P* < 0.01) with lower monocyte counts (Table 2, *P* < 0.05). Cortisol levels did not differ between the females and males, neither in cord blood (58.0 ± 2.1 vs. 63.4 ± 3.4 ng/mL, *P* > 0.1), at day 5 (87.5 ± 9.7 vs. 83.6 ± 7.5 ng/mL, *P* > 0.1) or by day 19 (60.0 ± 15.5 vs. 60.2 ± 18.2 ng/mL, *P* > 0.1). Furthermore, no differences in the serum biochemical parameters were found between female and male piglets on day 19, except higher aspartate aminotransferase levels in male piglets (Table 3, *P* < 0.05).

**Table 2:** Hematological parameters of female and male preterm pigs on day 1, 9, and 19.

	**Day 1**		**Day 9**		**Day 19**	
	**Female (*n* = 109)**	**Male (*n* = 116)**	**Female (*n* = 100)**	**Male (*n* = 102)**	**Female (*n* = 92)**	**Male (*n* = 95)**
Total leucocytes (10^9^ cells/L)	2.7 (0.0)	2.8 (0.0)	5.6 (0.3)	6.4 (0.3)^*^	9.9 (0.6)	9.5 (0.4)
Neutrophils (10^9^ cells/L)	0.6 (0.0)	0.6 (0.0)	3.6 (0.3)	4.1 (0.3)	7.1 (0.8)	6.2 (0.4)
Lymphocytes (10^9^ cells/L)	1.9 (0.0)	2.0 (0.1)	1.8 (0.1)	2.0 (0.1)	2.8 (0.1)	2.8 (0.1)
Monocytes (10^9^ cells/L)	0.06 (0.01)	0.05 (0.00)	0.13 (0.01)	0.15 (0.01)	0.38 (0.03)	0.31 (0.03)^*^
Eosinophils (10^9^ cells/L)	0.09 (0.01)	0.09 (0.01)	0.09 (0.05)	0.06 (0.01)	0.07 (0.01)	0.08 (0.01)
Basophils (10^9^ cells/L)	0.01 (0.00)	0.01 (0.00)	0.02 (0.01)	0.01 (0.00)	0.02 (0.00)	0.02 (0.00)
Platelets (10^9^ cells/L)	214 (8)	209 (9)	363 (20)	390 (22)	479 (20)	479 (22)
Red blood cells (10^12^ cells/L)	3.7 (0.0)	3.8 (0.0)	3.4 (0.1)	3.5 (0.1)	4.0 (0.1)	4.0 (0.1)
Hemoglobin (g/L)	5.2 (0.1)	5.2 (0.0)	4.4 (0.1)	4.2 (0.1)(^*^)	4.4 (0.1)	4.3 (0.1)^**^
Haematocrit (%)	28.1 (0.3)	28.1 (0.3)	23.8 (0.4)	23.2 (0.4)(^*^)	23.5 (0.5)	22.9 (0.5)^**^

Data presented as means with corresponding SE. ^(*)^Tendency to effect, P < 0.1,

* *P < 0.05*,

** *P < 0.01*.

**Table 3:** Serum biochemistry in female and male piglets at day 19.

	**Female (*n* = 95)**	**Male (*n* = 105)**
Albumin (g/L)	16.7 (0.3)	16.9 (0.3)
Total protein (g/L)	29.1 (0.5)	29.6 (0.6)
Alkaline phosphatase (U/L)	1,385 (81)	1,271 (69)
Alanine aminotransferase (U/L)	31.3 (0.8)	30.7 (0.8)
Total bilirubin (μmol/L)	2.0 (0.1)	2.3 (0.1)
Cholesterol (mmol/L)	2.7 (0.1)	2.7 (0.1)
Creatinine (μmol/L)	50.8 (1.1)	55.5 (2.1)
Creatine kinase (U/L)	241 (21.2)	245 (29.5)
Iron (μmol/L)	7.2 (0.6)	8.2 (0.5)
Phosphate (mmol/L)	2.1 (0.1)	2.1 (0.1)
Aspartate aminotransferase (U/L)	33.3 (1.4)	39.9 (2.7)^*^
Blood urea nitrogen (mmol/L)	3.7 (0.3)	4.5 (0.4)
Gamma-glutamyl transferase (U/L)	22.4 (0.9)	21.9 (0.9)
Calcium (mmol/L)	2.6 (0.0)	2.6 (0.0)
Magnesium (mmol/L)	0.9 (0.0)	0.9 (0.0)
Sodium (mmol/L)	142 (1.3)	143 (1.3)
Potassium (mmol/L)	4.4 (0.1)	4.4 (0.1)

*Data presented as means with corresponding SE*.

* *P < 0.05*.

**Figure 2:**
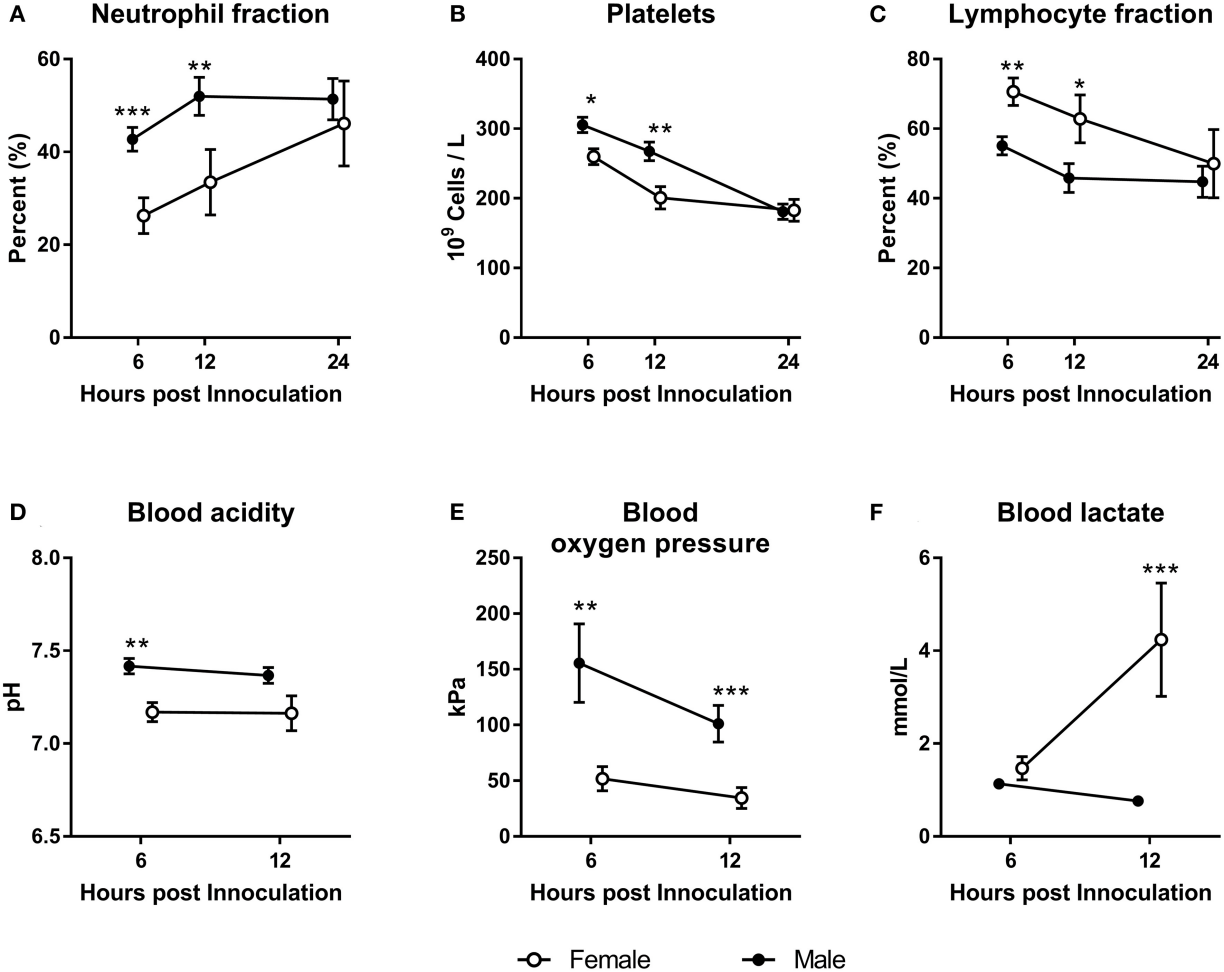
Hematological and blood gas parameters in female and male preterm pigs (open and filled circles, respectively) inoculated with live *S. epidermidis* bacteria shortly after birth. Neutrophil fractions (**A**, *n* = 38). Platelet counts (**B**, *n* = 38). Lymphocyte fractions (**C**, *n* = 38). Blood acidity (**D**, *n* = 38). Blood oxygen pressure (**E**, *n* = 38). Blood lactate levels (**F**, *n* = 38). All shown as means with corresponding SE. *P < 0.05, **P < 0.01, ***P < 0.001.

Animals inoculated with *S. epidermidis* right after birth showed marked sex-specific differences in their responses. Males showed higher neutrophil fractions and platelet counts with lower leucocyte fraction 6–12 h after inoculation (Figures 2A–C, *P* < 0.05–0.001). This result was coupled with a higher blood pH (Figure 2D, *P* < 0.05) and oxygen pressure (Figure 2E, *P* < 0.01–0.001), and with lower blood lactate (Figure 2F, *P* < 0.001). When the same experiment was conducted 48 h after birth, no differences in hematology or blood gas parameters between females and males were detected (data not shown).

**Figure 3:**
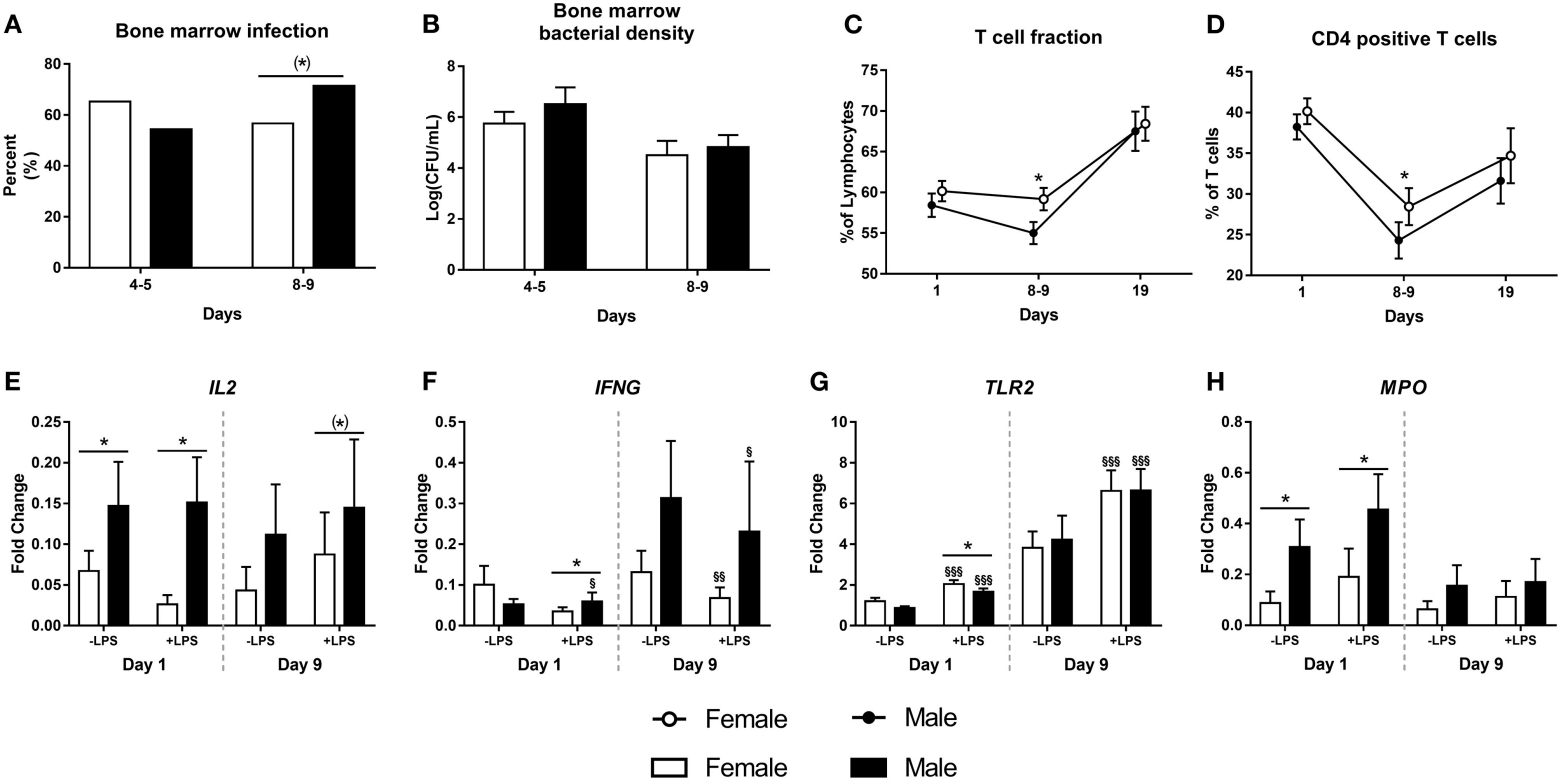
Incidence of bone marrow bacterial infection (**A**, *n* = 74 and 130) with corresponding bacterial densities (**B**, *n* = 74 and 130), as well as fractions of T cells (**C**, *n* = 52–148), CD4 positive T cells (**D**, *n* = 52–148) and expressions of *IL2, IFNG, TLR2*, and *MPO* (**E–H**, *n* = 38–81) in male and female preterm pigs. Shown as fractions **(A)**, means with corresponding error **(B–D)** and fold- changes in relation to housekeeping gene, before and after stimulation with lipopolysaccharide (LPS, **E–H**). (*)Tendency to an effect, *P* < 0.1, *P < 0.05. ^§^Effect of LPS, *P* < 0.1, ^§^*P* < 0.05, ^§§^*P* < 0.01, ^§§§^*P* < 0.001.

There was no difference in the occurrence of spontaneous bacterial infection in bone marrow on day 5, but by day 9, male piglets tended to have a higher infection incidence (71 vs. 56%, Figure 3A, *P* = 0.06). However, the bacterial densities in the bone marrow did not differ between females or males, neither at day 5 nor at day 9 (Figure 3B, both *P* > 0.1). There were no differences in T cell subsets at birth or by day 19. However, by day 9, male preterm pigs showed lower fraction of T cells and CD4 positive T cells (Figures 3C,D, both *P* < 0.05). Neutrophil phagocytic function did not differ between female and male preterm piglets at birth, day 9 or day 19 (data not shown).

Leucocyte gene expression at birth was performed for 81 preterm pigs (51% male) and was repeated at day 9 for a smaller subgroup (*n* = 38, 53% male). At birth, male piglets showed higher expression of *IL2*, both before and after stimulation with LPS (Figure 3E, both *P* < 0.05), male pigs also had higher expression of *IFNG* after stimulation with LPS (Figure 3F, *P* < 0.05). Furthermore, male piglets had lower expression of *TLR2* and with higher expression of *MPO* than females (Figures 3G,H, both *P* < 0.05). By day 9, there was a tendency to higher expression of *IL2* in male pigs after LPS stimulation (Figure 3E, *P* = 0.09). No further differences between female and male preterm pigs were found at day 9.

### Growth, Organ Weights and Gut Endpoints

**Figure 4:**
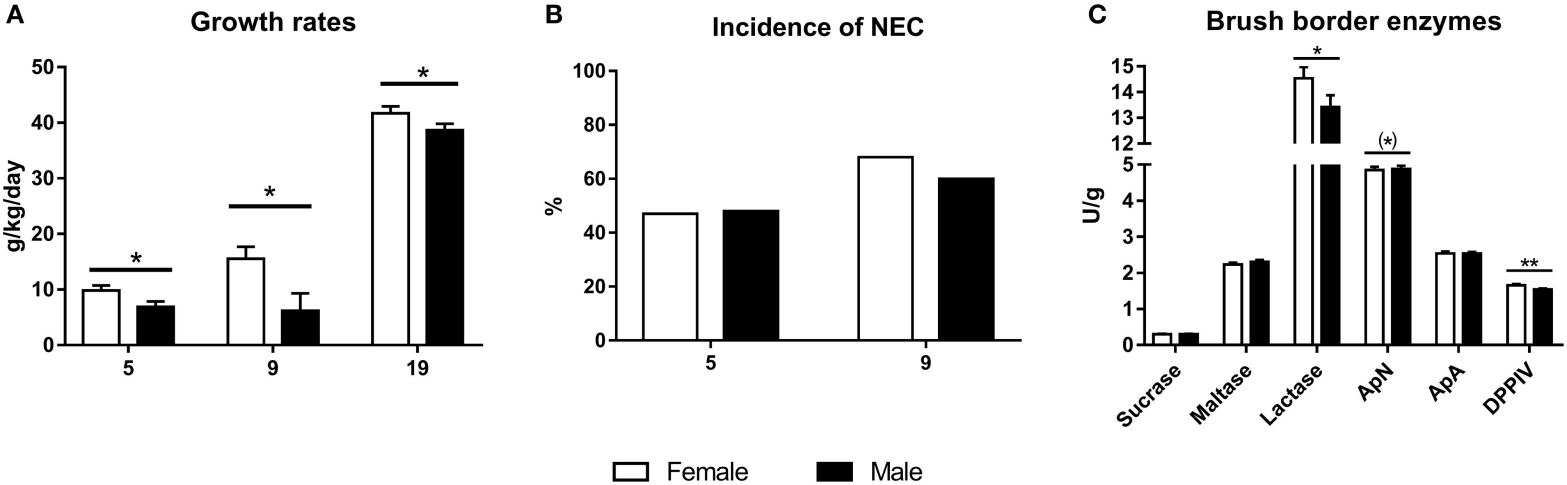
Growth rates for 5, 9, and 19 day cohorts (**A**, g/kg/day, *n* = 1,135, 319, and 221), incidence of necrotizing enterocolitis (NEC score ≥3) at day 5 and 9 (**B**, *n* = 1,152 for day 5 and *n* = 319 for day 9), and brush border enzyme activities at day 5 (**C**, *n* = 1,028) for male and female preterm piglets. Shown as means with corresponding SE **(A,C)** or as proportion of animals (%) in the cohort **(B)**. (*)Tendency to effect, *P* < 0.1, *P < 0.05, **P < 0.01.

Mean birth weight did not differ between female and male preterm piglets in any of the pig cohorts and ranged 912–998 g. However, relative daily weight gain was lower in male piglets, both at day 5, 9, and 19 (Figure 4A, all *P* < 0.05). For organ growth, 5 day male piglets showed lower relative kidney and small intestinal weight (Table 4, *P* < 0.001 and *P* < 0.05, respectively), with a tendency toward higher relative brain weight (Table 4, *P* = 0.065). In the cohort of pigs reared for 19 days, the relative kidney weight was still lower in male piglets (*P* < 0.001) whereas other organ weights did not differ, apart from a tendency to lower colon weight in male piglets both at day 9 and 19 (Table 4, *P* < 0.10). No sex-specific differences in body composition at day 19, as evaluated by DEXA scanning, were found (data not shown).

**Table 4:** Relative organ weights (g per kg body weight) of male and female preterm pigs.

	**Day 5**		**Day 9**		**Day 19**	
	**Female (*n* = 501–568)**	**Male (*n* = 457–514)**	**Female (*n* = 163)**	**Male (*n* = 156)**	**Female (*n* = 104)**	**Male (*n* = 116)**
Stomach	6.8 (0.1)	7.0 (0.1)	6.8 (0.2)	7.0 (0.3)	6.6 (0.1)	6.6 (0.1)
Small intestine	31.7 (0.3)	30.9 (0.3)^*^	32.5 (0.6)	33.8 (1.1)	40.2 (0.7)	39.5 (0.6)
Colon	12.2 (0.2)	11.9 (0.2)	16.4 (0.5)	15.5 (0.7)(^*^)	21.0 (1.3)	18.5 (1.3)(^*^)
Heart	8.1 (0.1)	7.9 (0.1)	7.5 (0.2)	8.0 (0.3)	7.2 (0.1)	7.3 (0.1)
Lungs	24.1 (0.3)	24.4 (0.3)	24.1 (0.6)	25.3 (0.9)	20.5 (0.6)	19.7 (0.6)
Liver	28.2 (0.2)	28.8 (0.2)	31.1 (0.7)	33.1 (0.8)^*^	25.7 (0.5)	25.5 (0.6)
Kidneys	10.6 (0.2)	10.0 (0.2)^***^	7.7 (0.2)	7.8 (0.3)	7.5 (0.1)	7.0 (0.1)^***^
Spleen	1.9 (0.0)	1.9 (0.0)	2.5 (0.1)	2.5 (0.1)	3.0 (0.1)	3.1 (0.1)
Brain^†^	28.3 (0.4)	29.9 (0.5)(^*^)	27.3 (0.8)	29.6 (1.1)	22.9 (0.6)	22.8 (0.7)

*Data are means with corresponding SE*.

† *On day 5, brains were collected from a subgroup (n = 615, 47% male)*. (*)Tendency to effect, P < 0.1,

* *P < 0.05*,

*** *P < 0.001*.

On day 5, 48% of pigs assessed for NEC (*n* = 1,152) showed mild-severe signs of NEC upon necropsy (score ≥3 in at least one region), while 36% showed more severe signs (score ≥4 in at least one region), but for both categories, no differences between males and females were observed (Figure 4B). Likewise, there was no difference in the average NEC severity score between males and females, even when stratifying across different regions of the gut (data not shown). The corresponding values for day 9 piglets with NEC scores (*n* = 319) were 64 and 54%, again with no differences between males and females.

After 5 days, male piglets showed lower activity of lactase and DPPIV (Figure 4C, *P* < 0.05 and *P* < 0.01, respectively) with a tendency to higher activity of ApN (*P* = 0.06). In pigs reared for 19 days, there were no differences in brush border enzyme activities (data not shown). Likewise, there were no difference between villus height or crypt depth across gut regions in pigs reared for either 5 or 19 days (data not shown).

### Neurodevelopment and Behavior

On day 19, male pigs had higher absolute weight of cerebellum (2.87 vs. 2.76 g, *P* < 0.05), and higher relative weights cerebellum (% of total brain weight, Table 5, *P* < 0.001), relative to female pigs. Other brain weight measures did not differ. Measured outcomes of motor function, explorative behavior, cognition, and visual memory (open field and T-maze-tests), did not show any significant differences between male and female piglets at any time points during the test (data not shown).

**Table 5:** Brain parameters in male and female preterm pigs after 19 days.

	**Female (*n* = 102)**	**Male (*n* = 115)**
Water content (%)	82.7 (0.1)	82.8 (0.1)
Cerebellum (%)	10.4 (0.1)	10.8 (0.1)^***^
Cerebrum (%)	79.9 (0.1)	79.7 (0.1)
Brainstem (%)	9.2 (0.1)	9.3 (0.1)
Hippocampus (%)	1.8 (0.2)	1.6 (0.0)
Striatum (%)	1.0 (0.0)	1.0 (0.0)

*Data are means with corresponding SE*.

*** *P < 0.001*.

## Discussion

To optimize the care and treatment of immature neonates, it is important to know to which extent males and females have different risk factors and respond differently to treatments. Using our preterm pig model of immature birth, we studied preterm male and female pigs during the neonatal transition (5 days), post-natal adaptation (9 days) and initial growth phases of development (19 days, Figure 1). Following elective cesarean section in late gestation, this animal model mimics many of the complications of weak, compromised piglets at term, and of very preterm infants (e.g., immature lung, metabolic, thermoregulatory, gut, immune, and brain functions (37, 38, 62), yet it avoids the possible confounding effects of fetal factors leading to preterm birth in humans (e.g., maternal inflammation, hypertension, placental dysfunction). Further, our standardized rearing and feeding protocols ensure that we can isolate intrinsic biological differences between the sexes, independent of interactions with their mother for thermoregulation, nutrient uptake or passive immunity. Using this model we now show that neonatal mortality in immature preterm pigs is much higher in male vs. female pigs, despite a seemingly improved resistance to bacterial infection within the first days. There was a clear reduction in body growth in surviving male pigs, but apart from this the observed sex-specific gut, immunity and brain differences were marginal, at least compared with effects of most nutritional, microbial, or pharmacological interventions in preterm pigs (37). How sex-specific differences shortly after preterm birth may develop toward puberty and adulthood remains to be shown, and in-depth studies on immunity, growth and organ functions are required in both pigs and infants.

Despite the clear difference in neonatal mortality, we are limited by the fact that our preterm pig studies did not include a detailed diagnosis of the cause of death. Yet, studies in infants suggest that poor respiratory function in preterm males is a key contributor to increased mortality (5) and cohort studies have shown that males, both late pre-terms and terms, have higher risk of respiratory distress syndrome (RDS) (63), possibly related to less surfactant production in late gestation (64, 65). Following cesarean section at 90% gestation, a large proportion of preterm pigs show RDS-like symptoms and lung immaturity, as assessed by reduced blood oxygen levels and macroscopic lung appearance at autopsy (atelectasis) (39, 41, 57, 61). However, we have not systematically registered the degree of post-natal respiratory distress in our studies. Interestingly, we did not detect any differences in cord blood levels of cortisol, a hormone well-known to stimulate lung development and respiratory function in preterm infants (66) although differences in cortisol production between the sexes may be masked by the cesarean section that may not stress the piglets as much as preterm labor. We have previously shown that blocking cortisol production in newborn pigs lead to increased neonatal mortality (29). Likewise, low cortisol levels at birth are associated with neonatal mortality in production pigs (30), highlighting the importance of this hormone after birth. Lower body temperature has also been observed in male production pigs and poor thermoregulation was suggested to contribute to increased mortality (16). However, we did not observe any differences in our study in the first 24 h of life but sex-specific differences in rectal temperature and thermoregulation could have been hidden by our tight control of temperatures in incubators.

Beyond the neonatal period, surviving male preterm pigs in our study showed reduced growth rate, kidney weight and activity of some digestive enzymes. Gut growth was not markedly affected although the slightly reduced intestinal weight on day 5, and tendency to reduced colon weight at day 9–19, may indeed reflect a slight delay in gut development in males. The magnitude of the transient male-specific reduction in lactase and DPPIV activity on day 5 remained quantitatively of lower magnitude (e.g., 5–10% reduction) than those of feeding formula vs. intact milk diets or colostrum (47, 67) or changes to gut bacterial colonization (relative to germ-free rearing or antibiotics treatment) (47, 55, 68). The limited effect of sex on gut development and NEC in preterm pigs, despite their high NEC sensitivity in the first 1–2 weeks (37), is consistent with observations in preterm infants, where gender does not markedly affect NEC risk (27, 69, 70).

The slower body growth in male vs. female preterm pigs is consistent with the finding that male preterm infants are at a higher risk to develop extra-uterine growth restriction (6, 7). Among internal organs, only kidney growth was consistently reduced in male preterm pigs. In preterm infants, glomerular filtration rate is similar in males and females (71) but males show a higher risk of acute kidney injury (72, 73). At 19 days, male pigs had slightly lower hemoglobin and haematocrit values than females, potentially related to diminished erythropoiesis by the smaller kidneys (74), but more studies are required to verify sex effects on renal structure and function. This organ could be particularly susceptible to perinatal stressors, as indicated by our recent studies on fetal inflammation on gut, lung, liver, immunity and kidney development in preterm pigs (33, 57, 75–77). In the literature on production pigs, males do not consistently show reduced neonatal survival and growth (18–20, 30), hence sex-specific survival, growth and adaptation may be most pronounced for immature newborns. Consequently, it may become increasingly relevant with sex-specific intensive care procedures for weak (immature) newborn pigs from hyperproliferative sows in modern pig production (e.g., resuscitation, cross-fostering, immunization, artificial rearing procedures, microbial protection).

Important sex-related differences were observed for some systemic immune endpoints just after birth, and these may interact with effects in internal organs. Despite that leucocyte or T cell subsets did not differ between preterm male and female piglets at birth, the leucocyte gene expression analysis showed that expression of genes encoding interleukin-2 (*IL2*) and interferon gamma (*IFNG*) were higher in males, also after stimulation with LPS. Both cytokines are important in development of a Th1-directed immune response (78). Together with a higher expression of myeloperoxidase (*MPO*) this may have made males more resilient to infection with *S. epidermidis*. Inoculation with the same bacteria later (48 h after birth) showed no differences between male and female piglets. However, the general response to infection was dampened from 2 to 3 days after preterm birth in pigs (23), potentially making sex-specific differences harder to detect from this age. A tendency to a higher rate of spontaneous infection from days 5 to 9 may indicate diminished immune function in males beyond the neonatal period. *Staphylococcus epidermidis* bacteria are considered pathogenic in preterm infants (79). Male preterm pigs also showed transiently lower T cell and CD4+ T cell fractions at day 9, but these disappeared by day 19, possibly reflecting age-related data from term 0 to 5 month old infants (80). We cannot exclude that species-specific differences in immune development between pigs and infants, including differential transfer of passive immunity (parentally via the placenta in infants vs. post-natally via colostrum uptake in the gut in pigs), affect our conclusions regarding sex effects. On the other hand, artificially-reared preterm pigs infused with maternal plasma to provide passive immunity (Figure 1) may very well reflect preterm infants normally born with low plasma levels of IgG and seldom receive mother's own milk or colostrum as their first enteral meals.

The only neurological parameter that differed between piglet sexes was the size of the cerebellum. While this may indicate better motor function, we saw no sex-related differences in the open field test, nor in the T-maze-test for visual memory capacity. In preterm infants, males have worse neurological outcomes following hypoxia, higher incidence of cerebral palsy and poorer long-term cognitive and language outcomes (81, 82), together with delayed myelination (83). The sex-differences in neurological outcomes are worse after more extreme pre-maturity (4). In this context, it is important to note that the brain, neurological outcomes and motor function are relatively mature in 90% gestation preterm pigs, relative to preterm infants, even if they show (temporary) post-natal deficits relative to term piglets (41, 43, 45, 60).

Across a large series of experiments with identical birth conditions (elective cesarean section) and clinically-relevant interventions in the same neonatal care facility, we aimed to mimic and standardize clinical responses to immaturity at birth for both pigs and infants. Still, it remains unclear how to translate results from preterm pigs to preterm infants because species similarities and differences are both age- and organ-dependent. Similarly, it remains speculative how well 90% preterm piglets reflect the clinical complications for the large proportion of normal term pigs dying during delivery or shortly after birth in modern pig production. The inclusion of a large number of endpoints from different cohorts increases the risk of false discoveries in statistical testing, despite large sample size. Regardless, our model provides a well-controlled and sensitive tool to test the effects of interventions and biological co-variants when individuals show immaturity at birth, and without the variable maternal interactions known to influence sex-specific survival rates in both pigs (16) and infants (1–4). Our results document that preterm pigs mimic many of the sex-specific differences in mortality, growth, and immune functions in preterm infants, supporting the use of this model to investigate sex-specific diseases of immature newborns. The mechanisms related to differences in immune response in the neonatal period, as well as the small effects on body and organ growth and function later (e.g., lung, gut, immunity, brain) warrant further investigations. At the population level, such biological differences may affect clinical outcomes but it remains questionable, if they justify sex-specific clinical treatment of immature and/or mature newborn individuals, either pigs or infants.

## Data Availability Statement

The raw data supporting the conclusions of this article will be made available by the authors, without undue reservation.

## Ethics Statement

The animal study was reviewed and approved by Danish National Committee on Animal Experimentation.

## Author Contributions

MC collected and organized the cohort data for analysis. OB analyzed data and produced the first draft of the manuscript. PS took responsibility for final editions in the text. All authors commented on the data, analyses and manuscript text, and approved the final version for submission.

## Conflict of Interest

The authors declare that the research was conducted in the absence of any commercial or financial relationships that could be construed as a potential conflict of interest.
