# Supplementary material for: Sex-Specific Survival, Growth, Immunity and Organ Development in Preterm Pigs as Models for Immature Newborns
Source: Front Pediatr. 2021 Feb 11;9:626101. doi: 10.3389/fped.2021.626101 (PMC7905020; doi:10.3389/fped.2021.626101)
Supplement: Supplementary file 1 [file Table_1.DOCX]

**Supplementary table 1: Overview of separate preterm piglet studies used to build 5-, 9- and 19-day cohorts and**

**investigate sex-specific effects on mortality, growth, organ, and immune parameters across studies**

| **Study** | **Length** | **Litters** | **Total** | **Female** | **Male** | **Study area and interventions** | **Reference** |
| --- | --- | --- | --- | --- | --- | --- | --- |
| **acronym** | **days** | **n** | **n** | **N** | **n** |  |  |
| 2FL 2012 | 5 | 2 | 44 | 22 | 22 | Milk diets - oligosaccharides | (1) |
| AB 2009 | 5 | 2 | 28 | 16 | 12 | Microbial - variable antibiotics | (2) |
| BioWPC 2014 | 5 | 4 | 60 | 33 | 27 | Milk diets - heat treatment | (3) |
| BioWPC 2013 | 5 | 2 | 31 | 18 | 13 | Milk diets - heat treatment | (3) |
| Cobed 2019 | 4 | 3 | 63 | 31 | 32 | Physiology - skin-to-skin contact | na |
| FFT1 2018 | 5 | 2 | 42 | 20 | 22 | Microbial - fecal filtrates | na |
| FFT2 2018 | 5 | 3 | 68 | 30 | 38 | Microbial - fecal filtrates | na |
| FMT 2015 | 5 | 3 | 58 | 31 | 27 | Microbial - fecal microbiota | (4) |
| FMT 2018 | 5 | 2 | 50 | 24 | 26 | Microbial - fecal microbiota | na |
| HMF 2016 | 5 | 4 | 80 | 36 | 44 | Milk diets - nutrient fortification | (5) |
| IGF1 2019 | 5 | 2 | 48 | 23 | 25 | Physiology - hormone replacement | na |
| LPSdiet 2017 | 5 | 5 | 67 | 34 | 33 | Milk diets - after fetal inflammation | (6) |
| MEN 2007 | 5 | 3 | 65 | 41 | 24 | Milk diets - slow enteral feeding | (7) |
| MixedMilk 2019 | 5 | 3 | 68 | 33 | 35 | Milk diets - milk-formula proportion | na |
| UHT 2019 | 5 | 3 | 61 | 36 | 25 | Milk diets - heat treatment | na |
| 25HMO 2013 | 5 | 2 | 53 | 15 | 38 | Milk diets – oligosaccharides | (8) |
| 4HMO 2012 | 5 | 4 | 44 | 19 | 25 | Milk diets – oligosaccharides | (8) |
| AB 2014 | 5 | 3 | 49 | 26 | 23 | Microbial - variable antibiotics | (9) |
| BioWPC 2012 | 5 | 4 | 61 | 34 | 27 | Milk diets - heat treatment | (10) |
| Cortisol 2015 | 5 | 2 | 38 | 19 | 19 | Physiology - hormone replacement | na |
| BAI 2014 | 5 | 2 | 50 | 23 | 27 | Physiology - bile acid inhibitor | na |
| DonorMilk 2009 | 5 | 3 | 40 | 22 | 18 | Milk diets - human and bovine milk | (11) |
| LPS 2016 | 5 | 3 | 31 | 16 | 15 | Microbial - fetal inflammation | (12) |
| MEN 2013 | 5 | 3 | 43 | 23 | 20 | Milk diets - slow enteral feeding | (13) |
| Probio 2007 | 5 | 3 | 39 | 21 | 18 | Microbial - dietary probiotics | (14) |
| RawMilk 2011 | 5 | 2 | 34 | 17 | 17 | Milk diets - processing effects | (15) |
| WPC 2010 | 5 | 5 | 83 | 35 | 48 | Milk diets - heat treatment | (16) |
| AB-FMT 2018 | 9 | 3 | 60 | 30 | 30 | Microbial - antibiotics/probiotics | na |
| HMF 2015 | 9 | 4 | 60 | 33 | 27 | Milk diets - nutrient fortification | (17) |
| IGF1 2019 | 9 | 3 | 68 | 35 | 33 | Physiology - hormone replacement | na |
| DonorMilk 2014 | 8 | 4 | 57 | 27 | 30 | Milk diets - human milk processing | (18) |
| MilkShift 2018 | 9 | 4 | 74 | 38 | 36 | Milk diets – milk/formula proportion | na |
| FortiColos 2017 | 19 | 2 | 34 | 15 | 19 | Milk diets - nutrient fortification | (19) |
| Alpha 2017 | 19 | 2 | 34 | 17 | 17 | Milk diets - protein composition | (20) |
| InfantBrain 2020 | 19 | 3 | 60 | 29 | 31 | Milk diets - lipid composition | na |
| OPN 2019 | 19 | 2 | 35 | 17 | 18 | Milk diets - bioactive ingredients | na |
| SAL 2015 | 19 | 3 | 58 | 26 | 32 | Milk diets - bioactive ingredients | (21) |

na, not availailable (sub-study results not published)

1. Cilieborg MS, Bering SB, Østergaard MV, Jensen ML, Krych Ł, Newburg DS, et al. Minimal short-term effect of dietary 2'-fucosyllactose on bacterial colonisation, intestinal function and necrotising enterocolitis in preterm pigs. *The British journal of nutrition* (2016) 116(5):834-41. Epub 2016/07/28. doi: 10.1017/s0007114516002646. PubMed PMID: 27452119.

2. Jensen ML, Thymann T, Cilieborg MS, Lykke M, Mølbak L, Jensen BB, et al. Antibiotics modulate intestinal immunity and prevent necrotizing enterocolitis in preterm neonatal piglets. *American journal of physiology Gastrointestinal and liver physiology* (2014) 306(1):G59-71. Epub 2013/10/26. doi: 10.1152/ajpgi.00213.2013. PubMed PMID: 24157972; PubMed Central PMCID: PMCPMC4073901.

3. Li Y, Østergaard MV, Jiang P, Chatterton DE, Thymann T, Kvistgaard AS, et al. Whey protein processing influences formula-induced gut maturation in preterm pigs. *The Journal of nutrition* (2013) 143(12):1934-42. Epub 2013/09/21. doi: 10.3945/jn.113.182931. PubMed PMID: 24047702.

4. Brunse A, Martin L, Rasmussen TS, Christensen L, Skovsted Cilieborg M, Wiese M, et al. Effect of fecal microbiota transplantation route of administration on gut colonization and host response in preterm pigs. *The ISME journal* (2019) 13(3):720-33. Epub 2018/10/28. doi: 10.1038/s41396-018-0301-z. PubMed PMID: 30367124; PubMed Central PMCID: PMCPMC6461782.

5. Sun J, Li Y, Pan X, Nguyen DN, Brunse A, Bojesen AM, et al. Human Milk Fortification with Bovine Colostrum Is Superior to Formula-Based Fortifiers to Prevent Gut Dysfunction, Necrotizing Enterocolitis, and Systemic Infection in Preterm Pigs. *JPEN Journal of parenteral and enteral nutrition* (2019) 43(2):252-62. Epub 2018/07/12. doi: 10.1002/jpen.1422. PubMed PMID: 29992630.

6. Ren S, Hui Y, Goericke-Pesch S, Pankratova S, Kot W, Pan X, et al. Gut and immune effects of bioactive milk factors in preterm pigs exposed to prenatal inflammation. *American journal of physiology Gastrointestinal and liver physiology* (2019) 317(1):G67-g77. Epub 2019/05/16. doi: 10.1152/ajpgi.00042.2019. PubMed PMID: 31091150.

7. Cilieborg MS, Boye M, Thymann T, Jensen BB, Sangild PT. Diet-dependent effects of minimal enteral nutrition on intestinal function and necrotizing enterocolitis in preterm pigs. *JPEN Journal of parenteral and enteral nutrition* (2011) 35(1):32-42. Epub 2011/01/13. doi: 10.1177/0148607110377206. PubMed PMID: 21224432.

8. Rasmussen SO, Martin L, Østergaard MV, Rudloff S, Roggenbuck M, Nguyen DN, et al. Human milk oligosaccharide effects on intestinal function and inflammation after preterm birth in pigs. *The Journal of nutritional biochemistry* (2017) 40:141-54. Epub 2016/11/28. doi: 10.1016/j.jnutbio.2016.10.011. PubMed PMID: 27889684.

9. Birck MM, Nguyen DN, Cilieborg MS, Kamal SS, Nielsen DS, Damborg P, et al. Enteral but not parenteral antibiotics enhance gut function and prevent necrotizing enterocolitis in formula-fed newborn preterm pigs. *American journal of physiology Gastrointestinal and liver physiology* (2016) 310(5):G323-33. Epub 2015/12/19. doi: 10.1152/ajpgi.00392.2015. PubMed PMID: 26680737.

10. Nguyen DN, Li Y, Sangild PT, Bering SB, Chatterton DE. Effects of bovine lactoferrin on the immature porcine intestine. *The British journal of nutrition* (2014) 111(2):321-31. Epub 2013/08/07. doi: 10.1017/s0007114513002456. PubMed PMID: 23915638.

11. Jensen ML, Sangild PT, Lykke M, Schmidt M, Boye M, Jensen BB, et al. Similar efficacy of human banked milk and bovine colostrum to decrease incidence of necrotizing enterocolitis in preterm piglets. *American journal of physiology Regulatory, integrative and comparative physiology* (2013) 305(1):R4-r12. Epub 2013/05/10. doi: 10.1152/ajpregu.00094.2013. PubMed PMID: 23657639.

12. Nguyen DN, Thymann T, Goericke-Pesch SK, Ren S, Wei W, Skovgaard K, et al. Prenatal Intra-Amniotic Endotoxin Induces Fetal Gut and Lung Immune Responses and Postnatal Systemic Inflammation in Preterm Pigs. *The American journal of pathology* (2018) 188(11):2629-43. Epub 2018/10/14. doi: 10.1016/j.ajpath.2018.07.020. PubMed PMID: 30314768.

13. Shen RL, Thymann T, Østergaard MV, Støy AC, Krych Ł, Nielsen DS, et al. Early gradual feeding with bovine colostrum improves gut function and NEC resistance relative to infant formula in preterm pigs. *American journal of physiology Gastrointestinal and liver physiology* (2015) 309(5):G310-23. Epub 2015/07/04. doi: 10.1152/ajpgi.00163.2015. PubMed PMID: 26138468.

14. Cilieborg MS, Thymann T, Siggers R, Boye M, Bering SB, Jensen BB, et al. The incidence of necrotizing enterocolitis is increased following probiotic administration to preterm pigs. *The Journal of nutrition* (2011) 141(2):223-30. Epub 2010/12/24. doi: 10.3945/jn.110.128561. PubMed PMID: 21178092.

15. Li Y, Jensen ML, Chatterton DE, Jensen BB, Thymann T, Kvistgaard AS, et al. Raw bovine milk improves gut responses to feeding relative to infant formula in preterm piglets. *American journal of physiology Gastrointestinal and liver physiology* (2014) 306(1):G81-90. Epub 2013/10/26. doi: 10.1152/ajpgi.00255.2013. PubMed PMID: 24157971.

16. Li Y, Nguyen DN, Obelitz-Ryom K, Andersen AD, Thymann T, Chatterton DEW, et al. Bioactive Whey Protein Concentrate and Lactose Stimulate Gut Function in Formula-fed Preterm Pigs. *Journal of pediatric gastroenterology and nutrition* (2018) 66(1):128-34. Epub 2017/07/29. doi: 10.1097/mpg.0000000000001699. PubMed PMID: 28753186.

17. Sun J, Li Y, Nguyen DN, Mortensen MS, van den Akker CHP, Skeath T, et al. Nutrient Fortification of Human Donor Milk Affects Intestinal Function and Protein Metabolism in Preterm Pigs. *The Journal of nutrition* (2018) 148(3):336-47. Epub 2018/02/21. doi: 10.1093/jn/nxx033. PubMed PMID: 29462356.

18. Li Y, Nguyen DN, de Waard M, Christensen L, Zhou P, Jiang P, et al. Pasteurization Procedures for Donor Human Milk Affect Body Growth, Intestinal Structure, and Resistance against Bacterial Infections in Preterm Pigs. *The Journal of nutrition* (2017) 147(6):1121-30. Epub 2017/03/17. doi: 10.3945/jn.116.244822. PubMed PMID: 28298536.

19. Ahnfeldt AM, Bæk O, Hui Y, Nielsen CH, Obelitz-Ryom K, Busk-Anderson T, et al. Nutrient Restriction has Limited Short-Term Effects on Gut, Immunity, and Brain Development in Preterm Pigs. *The Journal of nutrition* (2020) 150(5):1196-207. Epub 2020/02/19. doi: 10.1093/jn/nxaa030. PubMed PMID: 32069355.

20. Nielsen CH, Hui Y, Nguyen DN, Ahnfeldt AM, Burrin DG, Hartmann B, et al. Alpha-Lactalbumin Enriched Whey Protein Concentrate to Improve Gut, Immunity and Brain Development in Preterm Pigs. *Nutrients* (2020) 12(1). Epub 2020/01/23. doi: 10.3390/nu12010245. PubMed PMID: 31963562; PubMed Central PMCID: PMCPMC7020014.

21. Obelitz-Ryom K, Bering SB, Overgaard SH, Eskildsen SF, Ringgaard S, Olesen JL, et al. Bovine Milk Oligosaccharides with Sialyllactose Improves Cognition in Preterm Pigs. *Nutrients* (2019) 11(6). Epub 2019/06/19. doi: 10.3390/nu11061335. PubMed PMID: 31207876; PubMed Central PMCID: PMCPMC6628371.
